# Childhood cancer models of survivorship care: a scoping review of elements of care and reported outcomes

**DOI:** 10.1007/s11764-024-01610-6

**Published:** 2024-05-09

**Authors:** Natalie Bradford, Raymond Javan Chan, Xiomara Skrabal Ross, Carla Thamm, Erin Sharwood, Jason Pole, Christine Cashion, Larissa Nekhlyudov

**Affiliations:** 1 Cancer and Palliative Care Outcomes Centre and School of Nursing, Faculty of Health, Queensland University of Technology, Brisbane, QLD Australia; 2 Children’s Brain Cancer Centre at Centre for Children’s Health Research, 62 Graham St, South Brisbane, QLD Australia; 3 Caring Futures Institute, College of Nursing and Health Sciences, Flinders University, Bedford Park, SA Australia; 4 Research, Policy & Patient Department, Canteen Australia, Sydney, NSW Australia; 5 Endocrinology Department, Children’s Health Queensland Hospital and Health Service, South Brisbane, QLD Australia; 6 Queensland Digital Health Centre, Centre for Health Services Research, Faculty of Medicine, The University of Queensland, Brisbane, QLD Australia; 7 Department of Medicine, Brigham and Women’s Hospital, Harvard Medical School, Boston, MA USA

**Keywords:** Models of care, Survivorship, Paediatric, Cancer survivorship, Follow-up studies, Patient-reported outcome measures

## Abstract

**Purpose:**

This study aimed to systematically map elements of care and respective outcomes described in the literature for different models of post-treatment care for survivors of childhood cancer.

**Methods:**

MEDLINE, CINAHL, and Embase were searched with combinations of free text terms, synonyms, and MeSH terms using Boolean operators and are current to January 2024. We included studies that described post-treatment cancer survivorship models of care and reported patient or service level elements of care or outcomes, which we mapped to the Quality of Cancer Survivorship Care Framework domains.

**Results:**

Thirty-eight studies with diverse designs were included representing 6101 childhood cancer survivors (or their parent/caregiver) and 14 healthcare professionals. A diverse range of models of care were reported, including paediatric oncologist-led long-term follow-up, multi-disciplinary survivorship clinics, shared-care, and primary care-led follow-up. Elements of care at the individual level most commonly included surveillance for cancer recurrence as well as assessment of physical and psychological effects. At the service level, satisfaction with care was frequently reported but few studies reported how treatment-related-late effects were managed. The evidence does not support one model of care over another.

**Conclusions:**

Gaps in evidence exist regarding distal outcomes such as costs, health care utilization, and mortality, as well as understanding outcomes of managing chronic disease and physical or psychological effects. The findings synthesized in this review provide a valuable reference point for future service planning and evaluation.

**Implications for Cancer Survivors:**

Decades of research highlight the importance of survivorship care for childhood cancer survivors who are at risk of serious treatment-related late effects. This review emphasizes there is no single, ‘one-size fits all’ approach for delivering such care to this vulnerable population.

**Supplementary Information:**

The online version contains supplementary material available at 10.1007/s11764-024-01610-6.

## Introduction

Cancer in children is a relatively rare occurrence, but advancements in treatment and supportive care mean around 85% of diagnosed children in developed nations will become long-term survivors [1]. Despite this positive outcome, young survivors face a significant risk of treatment-related late effects, adversely impacting their long-term health and well-being. A growing body of literature highlights individuals diagnosed during childhood (0–14 years) experience significantly higher levels of morbidity and mortality compared to those diagnosed with cancer as older adults [1, 2].

Social, vocational, and educational milestones are also affected, leading to challenges in relationships and academic performance, and can result in social disadvantage and poorer mental health [3]. Moreover, late identification of adverse effects contributes to chronic disease, ultimately reducing life expectancy [4]. In response to these concerns, worldwide harmonized guidelines advocate for risk-based survivorship care across the lifespan to facilitate early detection and timely intervention to preserve health [5, 6].

To optimize long-term outcomes, quality survivorship care for this population requires a holistic approach encompassing multiple assessment and management domains. Quality survivorship care is defined as the ability to access effective healthcare structures and processes of care when needed [7]. In addition to surveillance for recurrence or new cancers, it is imperative to address the physical and psychosocial well-being of young survivors [8]. Acknowledging the unique needs of this vulnerable population, it is also essential to address their distinct needs regarding communication, information provision, decision-making, and care coordination. When these needs are recognized and accommodated, an age-appropriate and patient-centred experience is possible not only facilitating appropriate follow-up based upon individualized risk but also promoting continued adherence to long-term survivorship care [5].

Previous reviews have described the challenges of delivering equitable and coordinated survivorship care in this population, describing benefits and limitations of different models [9–11]. Models of paediatric oncologist-led long-term follow-up (LTFU) provide continuity of care and access to specialists, but place burgeoning demands on resources with the exponential growth of survivors requiring care [12]. Young survivors must transition at some point to adult-based care but options can be limited with unformalized processes and a lack of appropriate services to refer to [13]. Childhood cancer survivors and adult service providers may also be unaware of the long-term risks [13]. Primary care providers do not always receive the information required to deliver appropriate care to young cancer survivors [14], and are often not confident to do so [15].

To better understand the benefits of survivorship care, it is essential to comprehend the attributable outcomes. Previous reviews have mapped elements of survivorship care and outcomes for adult cancer populations against the Quality of Cancer Survivorship Care Framework [16]. The framework was developed through an evidence-based comprehensive, iterative process, in order to systematically deliver and evaluate the quality of cancer survivorship care [17]. The framework acts as a guide for the classification and categorization of survivorship care across the specified domains, which facilitates a structured synthesis, and has been utilized to assess survivorship interventions and outcomes in numerous systematic reviews [18–20].

The purpose of this scoping review was to synthesize elements of care and respective outcomes described in the literature for different models of post-treatment care for survivors of childhood cancer aged 0–14 years at diagnosis. These elements and outcomes are mapped to the domains of the Quality of Cancer Survivorship Care Framework [16].

## Methods

This review is reported according to the PRISMA extension for scoping reviews (Supplementary File 1). The protocol was prospectively registered with PROSPERO (CRD42022358713).

### Information sources

Electronic databases MEDLINE, CINAHL, and Embase were searched in September 2022 (updated January 2024) with combinations of free text terms, synonyms, and MeSH terms using Boolean operators (See Supplementary file 2 for search strategy). We supplemented our database search by reviewing the reference lists of included articles and key journals in the topic area.

### Eligibility criteria

Articles were eligible for inclusion if they met the criteria outlined in Table 1. Briefly, studies were required to report the following:

(i) Population: Individuals who were treated as a child (aged 0–14 years) for any type of cancer. We limited articles to age 14 years, as this is the age commonly reported for childhood cancer, and a corresponding review reports findings for those diagnosed with cancer as adolescents and young adults aged 15–39 years [21].
(ii) Intervention: (1) focussed on cancer survivorship, (2) include description of a model of care [22], and (3) report elements of care provided in the model
(iii) Context: We included articles published after January 2006, taking into account the seminal Institute of Medicine report “From Cancer Patient to Cancer Survivor: Lost in Transition” [23] published in 2005.
(iv) Outcomes: Patient and service level quality cancer survivorship elements of care and respective outcomes as described in Table 1.

**Table 1:** Eligibility criteria

*Population:* • Individuals who had completed planned primary cancer treatment for any type of cancer diagnosed as a child (0–14 years) • If articles included older adolescents and young adults, they were included in the study only if > 50% of the population were aged 0–14 years at diagnosis, or if data for that age group was separately available
*Intervention* 1. Cancer survivorship as the focus of the article. We defined this as the period following the completion of planned primary cancer treatment 2. Described a model of survivorship care. We defined a model of care as an overarching design for the provision of a particular type of healthcare, shaped by a theoretical basis, evidence-based practice and defined standards [22]. Models of care were grouped into categories (see Box 1) 3. Reported elements of care provided in the model
*Context:* • We included articles published after January 2006, taking into account the seminal Institute of Medicine report “From Cancer Patient to Cancer Survivor: Lost in Transition” [23] published in 2005
*Outcomes of interest:* • Studies that reported provision of elements of survivorship care and the respective outcomes, or barriers to care at the patient or service level. These were mapped to the Quality of Cancer Survivorship Care Framework [16] **Patient level** quality cancer survivorship elements of care and respective outcomes • Prevention and surveillance for recurrence and new cancers; management of physical and psychological effects; health promotion and disease prevention; management of chronic conditions; health-related quality of life/function and mortality **Service level** quality cancer survivorship elements of care and respective outcomes: • Clinical structure; communication/decision-making; care coordination and patient and caregiver experience; emergency services/hospitalizations and costs
*Types of studies:* • We included published empirical peer-reviewed studies, of any type of study design, except literature reviews, published in the English language beginning January 2006. We reviewed the full text of the literature reviews and included relevant primary studies

We excluded studies that focused on end-of-life care, did not include a description of the model of care, did not provide an evaluation or report on outcomes, or were published in 2005.

### Screening and abstraction

Following the searches, articles were collated in endnote and then exported to Rayyan for screening. To minimize bias, dual processes were used to select titles and abstracts and to screen full text following eligibility criteria (Supplementary file 3) by five authors (NB, CT, XS, LN, RC). Reasons for exclusion were noted, and discrepancies were resolved through discussion with a third author. The same authors then extracted data from included articles using a pre-defined data extraction sheet that was managed using Qualtrics. A second author (NB, CT, XS) checked the data abstraction of all articles.

### Synthesis

Studies were grouped into different models of care including long-term follow-up (LTFU) at treating cancer centre, specialist multidisciplinary care (e.g. survivorship clinics), shared care between primary care provider and paediatric oncologist, and ‘other’ various models (see Box 1). While critical appraisal is optional in scoping reviews, studies were graded against levels of evidence hierarchy to help inform the strength of the evidence (levels I–VII) [24]. We used a framework synthesis approach to analyse data, and a comparative method of thematic analysis using an organized structure across domains [25]. Matrices were developed to display the distribution and frequency of findings (elements of care and outcomes) across domains of Quality Cancer Survivorship Care Framework and reported barriers [16]. Through the process, findings were discussed with the authors to ensure validity and reliability. A narrative synthesis of findings is presented. To aid interpretation, the findings are presented under specified domain headings, highlighting those from the strongest design studies, including the description of the model of care, elements of care, and respective outcomes.

## Results

**Fig. 1:**
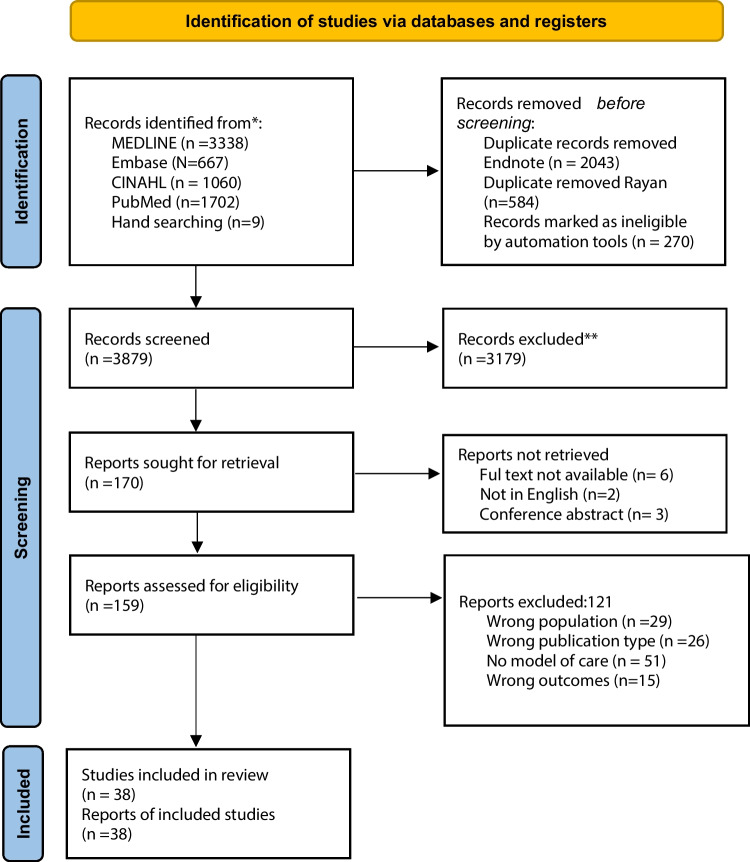
PRIMSA flow diagram. *From:* Page MJ, McKenzie JE, Bossuyt PM, Boutron I, Hoffmann TC, Mulrow CD, et al. The PRISMA 2020 statement: an updated guideline for reporting systematic reviews. BMJ 2021;372:n71. https://doi.org/10.1136/bmj.n71. For more information, visit: http://www.prisma-statement.org/

After duplicates were removed, the search identified 5298 articles. Of these, the full text was obtained for 179 articles and reviewed for eligibility. We included 38 primary studies representing 6101 childhood cancer survivors (or their parent/carer) and 14 healthcare professionals (Fig. 1).

There were two randomized controlled trials [26, 27] and six cohort studies that included a comparator group [28–33]. The remaining studies included 12 descriptive observational studies that followed a population of survivors over time [34–45], four retrospective cohort studies [46–49], eight cross-sectional survey studies [35, 50–56], five qualitative interview studies [57–61], and one used mixed methods with both survey and interview data [62]. Overall, as studies were mostly observational rather than experimental in nature, 58% were graded Level IV evidence [24] (Supplementary file). The median age across included studies ranged from 3 to 12 years at diagnosis and 13 to 37 years at time of study. The median time of follow-up ranged from 5 to 45 years post-diagnosis across different studies. Most (79%) articles were published after the year 2013 and 77% were in cancer-specific journals of which 42% were also paediatric cancer specific. Studies were from high-income nations (North America, Europe, Australia) with one study from Turkey [57]. Details of included study characteristics are outlined in the Supplementary Table.

### Models of care

Four studies directly compared outcomes from different models of care [26–28, 30]. One compared quality of life and physical symptoms from those attending paediatric LTFU at the cancer centre with primary care provider follow-up [30]. Another compared reports of late effects and satisfaction with LTFU provided by paediatric cancer centres with adult cancer centres [28]. One randomized controlled trial compared the efficacy of adherence to guideline recommended surveillance tests between a primary care led model supported with a survivorship care plan (SCP) and paediatric LTFU [27]. Another trial examined prevalence of post-traumatic stress disorder in a sub-group of randomized participants who had not previously attended any survivorship care [26].

Three population-based studies evaluated multiple models of care, describing different outcomes, commonly patient/carer attendance, and satisfaction [36, 50, 62]. One large (*n* = 3912) Canadian population–based study compared healthcare utilization outcomes between survivors who did and did not attend specialized survivorship clinics [31].

Ten studies described models of paediatric LTFU care, provided by the treating cancer centre with a focus on surveillance for cancer recurrence [37, 40, 43, 46, 48, 52, 55, 57, 58, 61]. Seven studies described a multidisciplinary model of care, provided through referral to a dedicated survivorship clinic [29, 39, 41, 42, 45, 47, 49]. These multidisciplinary models of care included coordinated access to in-person review/surveillance by multiple disciplines and subspecialties. Four studies described shared care models between primary care providers and paediatric specialist teams [32, 38, 53, 60]. Two studies described their model incorporating SCPs without elaborating how care was provided or who was responsible [32, 54]. The remaining studies included a joint paediatric/adult aftercare clinic model [35]; a multidisciplinary aftercare program based at the paediatric cancer centre offering uncoordinated referral to subspecialists [51]; primary care provider-only follow-up supported by a SCP [59]; a nurse-led survivorship clinic adjunct to paediatric oncologist LTFU [33]; a school liaison program [63]; a multi-disciplinary telehealth delivered survivorship intervention [34]; and a neuropsychology clinic for non-CNS diagnoses [44]. Further details are available in Table 2. Elements of care and the respective outcomes reported in the various models of care are detailed in the summary tables and figures below.

**Table 2:** Models of care and respective reported outcomes

Individual Level	**Quality domain**	**Findings (quantitative and *qualitative*)**	**Statistically significant findings**
	**Model of care**	**Recurrences and new cancers**	
	Paediatric specialist versus primary care model		↑Adherence to recommendations in pediatric specialist model [27, 30]
	Long-term follow-up versus population control	30–95% do not attend follow-up [37, 39, 48, 49, 62]. Those who do attend 55–82% aware of risks [29]. Up to 29% may be eligible for genetic counselling [41] ↑attendance for cardiomyopathy screening	
	Joint paediatric/adult aftercare clinic model	5 year lost to follow-up 3.8% [35]	
	Multidisciplinary distance-delivered intervention	↓Adherence to recommendations over time [34]	
	Shared care		71% of survivors adhere to recommendations[38] ↓Lost to follow-up [32]
		**Physical effects**	
	Paediatric specialist versus primary care model		↓Symptom burden [30]
	Paediatric versus adult specialist cancer centre	No difference in symptom burden [28]	
	Long-term follow-up vs population control	No difference in physical function [29]	
		**Psychological effects**	
	Paediatric versus adult specialist cancer centre	No difference in vulnerability [28]	
	Multidisciplinary survivorship clinic	50% classified as PTSD likely [26] No significant differences between those who do, and who do not attend follow-up care [29]	Significant minority report psychological or emotional problems, 4.2–6.9% evidence of PTSD [29]
	Joint paediatric/adult aftercare clinic model	High cancer worries, good self-management skills [35]	
	Long-term follow-up	*Need for ↑ psychological support *[57]	
		**Health-related quality of life**	
	Paediatric specialist versus primary care model	No difference in health-related quality of life [30]	
	Long-term follow-up vs. population control	No difference in health-related quality of life [29, 36, 55]	
	Multidisciplinary survivorship clinic	Assessed 41% of survivors as below population norms [42]	
		**Health promotion**	
	Long-term follow-up	*More information on health promotion wanted *[57] *Most felt hospital-based healthcare was the best place to receive healthy lifestyle advice but fewer than 10% wanted dietary or physical activity advice. *[61]	
	Provision of survivorship care plan	66% reported modifying health behaviours [54]	
		**Chronic conditions**	
	Paediatric versus adult specialist cancer centre	No differences in prevalence of chronic conditions[28]	
	Long-term follow-up	Prevalence of any chronic health condition 64–82% with 40% having more than 3 [40, 46]	
	Shared care	*Perceived lack of knowledge of late effects among survivors and primary care providers*[60]	
Health Service levl		**Clinical structure**	
	Paediatric versus adult specialist cancer centre	No differences in wait times [28]	
	Multidisciplinary coordinated model	↓Wait times [45]	
	Long-term follow-up vs population control	Lack of awareness main reason for non-attendance [29]	
	Long-term follow-up	Improvements needed in MDT processes and education [52] *Clinic efficiency and parking are barriers to attendance *[58]	
	Neuropsychologist follow-up	Implementation of the clinic feasible, 75% of patients passed screening and low conflict with algorithm and clinical judgment [44]	
		**Communication/decision-making**	
	Paediatric specialist versus primary care model	No significant difference in use of treatment summaries [30]	
	Paediatric versus adult specialist cancer centre		Number of topics discussed in pediatric model [28]
	Nurse-led clinic supported by oncologist		↑Awareness of late effects [33]
		**Care coordination**	
	Long-term follow-up	Contact with primary care provider ↑adherence to screening. Oncologist initiates 71% of discussion. [43]	
	Survivorship care plans	92% shared SurPass with primary care provider [54] 97% planned to share Survivorship Care Plan with primary care provider but only 60% did so. All caregivers agreed the plan would help make decisions about their child’s future care [63]	
	Primary care provider with survivorship care plan	*Survivorship Care Plan* *is an informative resource supporting communication and empowering survivors. Majority prefer web-based *[59]	
	Shared care	*Dissatisfaction with care due to poor coordination oncologists were preferred care providers* [60]	
		**Patient/caregiver experiences**	
	Paediatric versus adult specialist cancer centre	No difference in satisfaction between paediatric/ adult model of care [28]	
	Long-term follow up	More psychoeducation required [51]. Survivors attending long-term follow-up more likely to understand risks [33]. More than 90% satisfied with care [46] *93% satisfied but 40% wanted more details about long-term effects. *[61]	
	Multiple models	60% reported no follow up care 42% missed this with 30% dissatisfied [50]	
	Shared care	Patients report increased knowledge and satisfaction with care [53]	
	Primary care provider with survivorship care plan	*Barrier is a lack of confidence in primary care provider knowledge *[59]	
	School liaison program	*Perceived improvements to academic performance, home school communication, and school understanding of needs *[63]	
	Survivorship care plans	70% of caregivers thought the Survivorship Care Plan should be provided at end of treatment [56]	
		**Healthcare utilization**	
	Long-term follow-up	Estimated numbers of hospital admissions/ambulatory care, but may not be attributable to survivorship care [47]	
		**Mortality**	
	Multi-disciplinary survivorship clinic	Estimated at 17% for all time, but 3% when observing 5-year disease free survival [47]	

### Elements of care and respective outcomes

**Fig. 2:**
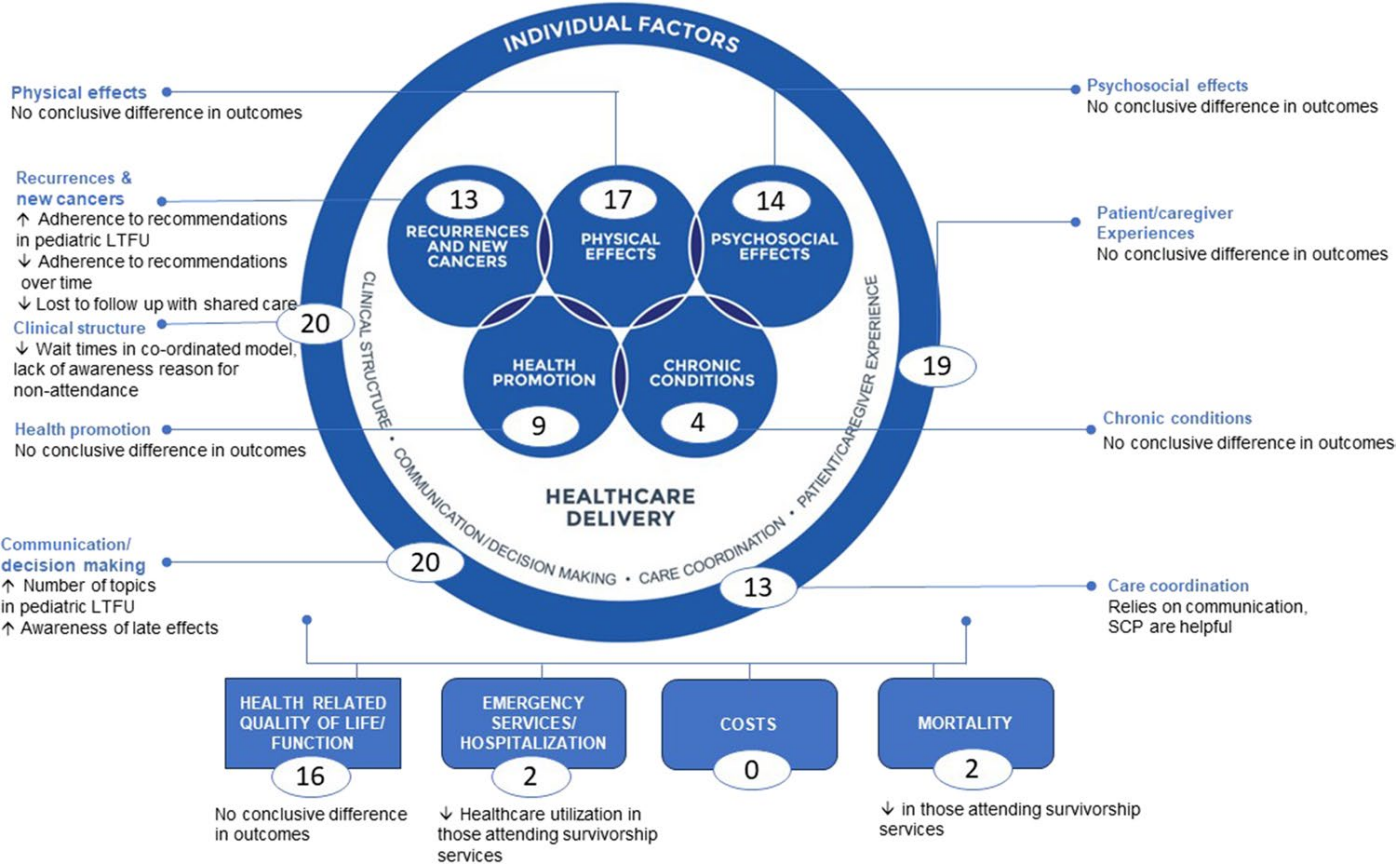
Count of studies reporting on elements of care or outcomes of the Framework for Quality Cancer Survivorship

Figure 2 provides a summary of the count of elements of care that map to the Quality of Cancer Survivorship Care Framework domains for each study. The elements of care and outcomes reported in each study are summarized in a matrix (Table 3).

**Table 3:** Elements of care or outcomes reported in each study

Author [ref]	Model of care	Patient level elements and outcomes						Healthcare service level elements and outcomes					
		Recurrence and new cancers	Physical effects	Psychological effects	Health promotion	Chronic conditions	Quality of life	Clinic structure	Communication/decision-making	Care coordination	Patient & caregiver experience	Emergency services/hospitalization	Mortality
Absolom et al. [25]	Paediatric vs adult LFTU	✔	✔	✔		✔	✔	✔	✔		✔		
Alchin et al. [31]	MDT telehealth			✔					✔	✔	✔		
Arpaci et al. [54]	LTFU				✔						✔		
Arvidson et al. [47]	Multiple models								✔		✔		
Aukema et al. [48]	After care		✔	✔			✔		✔	✔	✔		
Babecoff et al. [43]	LTFU	✔	✔			✔	✔				✔		
Berger et al. [35]	Shared care					✔	✔	✔	✔	✔	✔		
Cacciotti et al. [49]	LTFU	✔	✔				✔	✔		✔	✔		
Carlson et al. [42]	MDT/survivorship clinic	✔	✔	✔			✔	✔	✔	✔	✔		
Costello et al. [50]	Shared care		✔					✔	✔		✔		
Daly et al. [36]	MDT/survivorship clinic	✔											
Ducassou et al. [29]	Shared care		✔						✔	✔			
Ernst et al. [59]	Multiple models		✔						✔				
Ford et al. [26]	MDT/survivorship clinic		✔	✔	✔		✔		✔				
Gandy et al. [55]	LTFU			✔	✔			✔	✔	✔	✔		
Gebauer et al. [37]	LTFU	✔	✔			✔		✔					
Harper et al. [44]	LTFU						✔					✔	✔
Haupt et al. [51]	SCP			✔	✔				✔		✔		
KadanLottick et al. [24]	SCP + PCP vs LTFU	✔	✔										
Kam et al. [32]	LTFU-joint paediatric/adult	✔					✔		✔		✔		
Keats et al. [56]	PCP	✔	✔	✔	✔			✔	✔	✔	✔		
Knapke et al. [38]	MDT/survivorship clinic							✔		✔			
Lie et al. [57]	Shared care							✔		✔	✔		
Lindell et al. [30]	Nurse-led clinic		✔				✔		✔				
Linendoll et al. [39]	MDT/survivorship clinic		✔	✔	✔	✔	✔	✔	✔	✔	✔		
Marr et al. [45]	LTFU	✔						✔					
Mayes et al. [58]	LTFU				✔			✔	✔				
Meeske et al. [52]	LTFU		✔				✔	✔					
Mellblom et al. [40]	LTFU		✔	✔				✔	✔				
Michel et al. [33]	Multiple models		✔	✔			✔	✔			✔		
Northman et al. [60]	School program			✔				✔		✔	✔		
Ou et al. [46]	MDT/survivorship	✔						✔					
Pannier et al. [53]	SCP				✔						✔		
Reynolds et al. [27]	PCP vs LTFU	✔			✔		✔	✔		✔			
Ross et al. [23]	PCP + SCP vs LTFU			✔			✔						
Vetsch et al. [34]	LTFU	✔							✔				
Whitaker et al. [41]	Neuropsychology follow-up			✔			✔	✔	✔				

*SCP* Survivorship Care Plan, *LTFU* long-term follow-up, *MDT* multidisciplinary clinic, *PCP* primary care provider

### Patient level

#### Prevention and surveillance for recurrent and new cancers

Explored in 13 (34%) studies, prevention and surveillance for new cancers were common across all models of care, particularly with specialist-led (paediatric or adult) models of LTFU. Adherence to guideline recommendations for surveillance tests were significantly higher in follow-up at specialist cancer centres [48] when compared with primary care models [27, 30].

In descriptive studies, 71% of survivors reported adherence to recommendations in a shared care model with joint consultation between internists, patients, and primary care providers [38]. This contrasts with a cohort study of a telehealth delivered intervention that provided an average of seven recommendations per survivors, but only two were recalled by survivors at 6 months, with adherence to a mean of one recommendation [34]. In other descriptive studies, including population-based cohorts, between 30 and 95% of survivors were reported as not attending any kind of follow-up care [37, 39, 49, 62]. Predictors of not attending follow-up care were identified in two studies and included those survivors who received surgery or radiation only, older age, black or multi-race, and those who lived > 25 miles from the clinic [39, 49]. Other reported individual barriers to survivorship care included lack of understanding about the purpose of follow-up, lack of parental involvement, and motivational aspects on behalf of the survivor [39, 59, 62]. Recurrence or new cancers were reported in 3–10% of survivors during study periods [40, 47].

#### Surveillance and management of physical effects

Two studies compared the prevalence of physical symptoms in different models of survivorship care [28, 30]. One cohort study (*n* = 156) compared survivors attending paediatric LTFU with a primary care model, reporting those attending paediatric LTFU had 0.4–7.1% fewer physical symptoms [30]. In the second cohort study (*n* = 198), there were no differences in the prevalence of symptoms observed between paediatric versus adult LTFU care [28]. Prevalence of late effects or chronic conditions resulting from cancer treatment are reported below.

#### Surveillance and management of psychosocial effects

Three studies reported on psychosocial effects [29, 44, 54]. One cohort study (*n* = 173) compared psychological outcomes between survivors who did, and who did not attend LTFU [29]. While up to 19% of survivors in this study reported post-traumatic stress disorder or psychological distress, there were no significant differences between groups for these or other domains including: optimism, post-traumatic growth, and fear of recurrence, posttraumatic stress disorder [29]. In a cross sectional study (*n* = 190), around 30% of survivors reported some increase in their anxiety related to possible health consequences upon receiving SCP/treatment summaries [54]. The third study was a descriptive study (*n* = 215) that examined the feasibility of implementing neuropsychological screening for all survivors in their model of survivorship care, which deemed the initiative successful with only 25% of patients requiring further evaluation [44].

#### Health promotion and disease prevention

Four studies described health behaviours or promotion as outcomes of their models of care [29, 33, 54, 61]. Two cohort studies matched survivors who did and who did not attend LTFU; no significant differences in terms of health behaviours including use of alcohol, sunscreen, and physical activity were identified in one study (*n* = 173) [29], but survivors who attended LTFU were more aware of their risks for late effects in the other study (*n* = 174) [33]. A cross sectional study (*n* = 190) reported 67% of survivors who received a SCP/treatment summary reported recommended modifications to their lifestyle [54]. One qualitative study (*n* = 51) identified health promotion was not always considered a purpose of their LTFU with only 14% of survivors expecting to receive lifestyle advice while attending; 90% believed healthy lifestyle information was general knowledge attained elsewhere [61].

#### Management of chronic conditions

Assessment and management of chronic conditions were infrequently described with only two studies reporting prevalence of these outcomes [40, 46]. A descriptive study (*n* = 220) reported 64% of survivors attending a LTFU clinic were estimated to have at least one chronic health condition, and 30% of those had three or more [40]. Another retrospective cohort study (*n* = 51) of a LTFU clinic estimated 82% of survivors had at least one chronic disease, and again 30% had three or more [46]. The mean delay from diagnosis to onset of chronic disease post-treatment was 9.8 years, and authors reported concerns of underestimation of risk based on risk stratification guidelines [40]. Prevalence of chronic conditions identified in these two studies included endocrine (9–19%), neurologic impairment (14–17%), metabolic changes (16%), orthopaedic (9–13%), renal (9%), and cardiac conditions (9%) [40, 46]. Neither study included their approach or effects of management.

#### Health-related quality of life/function

Seven studies reported health-related quality of life or function of survivors. One cross-sectional study (*n* = 86) of a LTFU clinic highlighted the unique position of LTFU to monitor health-related quality of life over time for childhood cancer survivors and reported that overall health-related quality of life was similar to that of the general population [55]. However, they also reported prevalence of fatigue and late effects correlated with poorer physical functioning [55]. This concurs with a descriptive study (*n* = 112) of LTFU that identified physical and psychological domains of quality of life of survivors were comparable to population norms [36] but contrasts with another descriptive study (*n* = 144) from a multi-disciplinary survivorship clinic that reported 41% of their cohort were below population norms for health-related quality of life [42]. Another small cross-sectional study (*n* = 22) of LTFU reported using quality of life measures (specifically the PedsQL Brain Cancer Module) to make improvements to their survivorship care program, including increased education and participation from multi-disciplinary team members [52]. No difference in health-related quality of life between survivors was observed in two cohort studies that compared LTFU at a paediatric cancer centre with adult specialists or primary care [28, 30]. Another study that compared survivors who did, and did not attend LTFU found no significant differences in current health status [29].

#### Mortality

Mortality was only reported in two studies [31, 47]. One large retrospective cohort study (*n* = 1379) of a Canadian multi-disciplinary survivorship clinic estimated mortality was 17% of all survivors, although when this period was restricted to only 5 years this reduced to 3% [47]. The second study was also a large cohort study (*n* = 3912) from Canada that calculated mortality at 0.71% of survivors who attended a survivorship clinic at least once, compared to 1.8% who never attended during the observation period [31].

### Service level

#### Clinical structure

Clinical structure was a commonly reported element of models of survivorship care described in eight studies. One cohort study (*n* = 198) compared paediatric specialist versus adult specialist LTFU, wait time was perceived as longer, and consultation time shorter in the adult model [28]. A cross-sectional study (*n* = 73) described a joint paediatric/adult clinic where oncologists work together to provide a continuous model of care [35]. While acknowledging the resource-intensive demands of this model, this study had a lost to follow-up rate of just 4% compared to higher rates (up to 95%) reported in other studies [39, 62]. Only one descriptive study (*n* = 66) provided estimates of the duration of LTFU consultations, which were on average 24 min (range 5–49) [43].

A cohort study (*n* = 72) found the intervention of shared care significantly decreased lost to follow-up cases [32]. One descriptive study (*n* = 57) described the initiation of a hospital-based school liaison program primarily for children with brain cancer and leukaemia which parents perceived as helpful for improving knowledge and advocacy with the development of formalized plans for neurocognitive late effects [63].

Qualitative studies (*n* = 27–34) highlighted the importance of efficient clinical operations, parking, and rapport with providers and survivors stressed the importance of having a family member present as an enabler [58, 60]. Other service level barriers included termination of services by the health care provider and structural barriers such as distance [39, 59, 62].

#### Communication/decision-making

Communication and decision-making were reported in nine studies. The use of treatment summaries was similar between paediatric LTFU and primary care in a cohort study (*n* = 156), with both models relying on these to organize follow-up surveillance tests [30]. Another cohort study (*n* = 198) reported a greater number of topics were discussed in paediatric versus adult LTFU (3.72 vs 5.36, *p* < 0.001) [28], which was report to empower survivors. This study reported electronic/web-based SCP were likely preferable to young cancer survivors and the inclusion of a timeline and personalized lifestyle information were highlighted as important components [59]. In a small cross-sectional study (*n* = 19), shared care using telehealth made it easier for the survivors to communicate about their cancer to their primary care provider and 94% reported they were also confident their primary care provider could address their needs [53]. In a descriptive study (*n* = 150) also evaluating a shared model of care, 77% of primary care providers reported they were previously poorly informed about their patient’s long-term risk of complications and 88% appreciated the joint consultation and documentation, with 71% of survivors subsequently reporting following recommendations [38].

In another small cross-sectional study (*n* = 21) specifically evaluating SCPs, most (90%) agreed it was a valuable tool that contained all the information required. Most (70%) parents thought the SCP should be provided soon after treatment, and while 95.7% of parents intended to share their child’s SCP with another provider, family, or school, only 60.9% did so (*P* < 0.01) [56]. In a descriptive study (*n* = 112) describing survivor expectation in consultations reported most commonly expecting to discuss their current health status and late effects during; the majority of late effects raised in LTFU consultations were in the context of current symptoms rather than future risk [36]. In another descriptive study (*n* = 66) of a LTFU clinic oncologists initiated 70% of physical, 51% of psychosocial, 97% of routine screening, and 90% of lifestyle discussions with parents and survivors initiating the balance [43]. Lack of awareness was reported as a major barrier for attendance at multidisciplinary survivorship clinic in a cohort study (*n* = 173); 71% of survivors reported not knowing services existed [29].

#### Care coordination

Two studies reported aspects of care co-ordination. Parents of child survivors of brain cancer reported in a cross-sectional study (*n* = 42) that their aftercare needs were mostly met, although they needed to self-refer for some services and most reported a need for improvement about timeliness of care and psychoeducation [51]. Coordinating care through a multidisciplinary survivorship clinic was anticipated to reduce rates of failure to follow-up on a referral in one cross-sectional study (*n* = 130). Other reported benefits were the ability to immediately access inter-disciplinary subspecialists on the one day in the one location [45].

#### Patient and caregiver experience

Patient and caregiver experience as reported through assessment of satisfaction in seven studies. No differences in survivor’s satisfaction were identified a cohort study (*n* = 198) comparing paediatric and adult LTFU [28]; however, a population-based cross-sectional study (*n* = 245) reviewing various models of care [50] found 38% were dissatisfied with follow-up because of follow-up being discontinued, lack of psychological follow-up, and dissatisfaction with transition to adult care. Survivors who were followed up at paediatric specialist centres reported in a descriptive study (*n* = 130) were satisfied (83%), with 97% reporting they would recommend it [45]. Similarly, a small cross sectional study (*n* = 22) found satisfaction was high with aftercare for brain cancer survivors provided through a paediatric specialist centre with most families reporting they received high-quality care [52]. In one shared care model reported in a small cross sectional study (*n* = 19), 94% of both survivors and primary care providers reported they would participate in other telehealth visits [53]. The use of SCP/ treatment summaries were also positively received in another larger cross sectional study (*n* = 190) with 98.4% of survivors agreeing there were benefits [54]. A qualitative study (*n* = 34) also supported shared care models to meet the unmet needs of survivors [60]. A descriptive study (*n* = 112) exploring survivor preferences for model of care found survivors rated satisfaction with oncologist-led consultations higher (4.06, 95% CI 3.91–4.22) than nurse (3.30, 95% CI 3.11–3.49), primary care provider (2.68, 95% CI 2.45–2.91), or postal/telephone (2.69 95%CI 2.47–2.91) although 30% rated all four models as equally preferable [36].

#### Emergency services/hospitalizations

Two studies reported on the use of healthcare utilization [31, 47]. Up to 94% of survivors followed in one large retrospective study (*n* = 1379) had a mean of eight hospital discharges per survivor in the study period, a mean of 192 ambulatory care events observed, and appeared in a mean of 203 practitioner claims; the authors note some of these events may have occurred during the treatment phase and that not all may be related to their cancer diagnosis [47]. The second large population–based cohort study (*n* = 3912) [31] identified emergency presentation visits were 19% lower for individuals who attended survivorship clinics compared to those who did not, and moreover, the rate of low-acuity emergency visits was significantly lower (relative rate 0.76, *p* < 0.001) [31].

#### Costs

No studies specifically reported on costs, either from the health service or the individual perspective. A cohort study (*n* = 156) that compared paediatric LTFU with primary care models reported the clinical structure was determined by available funding with high costs associated with cancer centre models contributing to decisions for discharge to primary care [30]. A barrier posed for shared care models identified in a cross-sectional study (*n* = 19) was the additional time (and thus costs) required for a primary care provider to manage complex patients [53]. However, in one cohort study (*n* = 72) of a shared care model, only 46% of primary care providers requested the forms to claim extra reimbursement, suggesting finances were not a driving factor for participating in the model of care [32].

## Discussion

Our aim in this scoping review was to synthesize elements of care and outcomes reported across studies examining models of childhood cancer survivorship care, mapped to the Quality Cancer Survivorship Care Framework [16]. As most of the studies were observational, we were not able to examine the effectiveness of the models of care. The included studies describe heterogenous models, with disparate structures and processes. Common between models is the medically driven nature of care, predominantly delivered in paediatric settings, although adult-based care and primary care settings were also represented. Multidisciplinary survivorship clinics, including a nurse-led survivorship clinic [33] and use of SCP for communication and information exchange are also described. Across these diverse models, a broad range of elements of care were identified with most domains of the framework represented, though few reported on measured outcomes of care. No model of care described more elements of care or outcomes than any other. However, across the 13 possible elements of care and outcomes, most (68%) reported on less than five of these. The domains most represented were elements of care rather than outcomes at the service level such as the clinic structure (52%), communication and decision-making (52%), and patient/carer satisfaction (50%). At the patient level, quality of life outcomes and assessment and management of physical symptoms were represented in 40% of studies. There were few studies reporting on health promotion and disease prevention (23%), management of chronic conditions (13%), mortality and healthcare utilization (5% each),and none reported on costs. Given the accumulating evidence suggesting a significant proportion of childhood cancer survivors will experience severe or life threatening chronic health conditions, survivorship services should prioritize supporting more health promotional activities and measure these outcome [64].

### Effectiveness of models of care

The evidence reviewed does not equivocally support one model of care over another. Few studies employed methods robust enough to provide a high level of evidence to support findings. In those that do, the evidence is not definitive. For example, while paediatric LTFU shows higher adherence to recommendations compared with primary care models [27, 30], other studies suggest a shared care model increased adherence [38, 43] and decreases rates of lost to follow-up [32]. Moreover, a lower symptom burden observed in paediatric LTFU compared with primary care models does not translate to significant impacts on health related quality of life raising questions about the clinical significance of findings [30].

Risk-driven guidelines form the basis for all models of childhood cancer survivorship care and studies in this review, and others suggest adherence to such guidelines were significantly higher in follow-up at specialist cancer centres when compared with primary care models [48]. This contrast with studies from adult cancer survivors where primary care models demonstrated as high, or more surveillance as specialist led care [18]. These differences may be due to the preparedness of primary care providers to deliver tailored survivorship care childhood cancer survivors; a substantial barrier identified in systematic reviews [14]. The studies in this review that examined shared care or primary care reported positive outcomes when primary care providers were supported to deliver care with risk-informed SCP [59], joint telehealth consultations [34, 53], and models involving specialist, primary care provider, and survivor preferences for care [38].

In many countries, childhood cancer survivors predominantly receive care from their primary care provider, with limited access to specialist led care as they transition to adulthood [12]. However, there is a notable gap in the literature regarding the management of this transition, or how survivors themselves perceive this process [65]. Recognizing the importance of knowledge and education for both providers and patients, and integrating these into all models of care is essential [65]. While different models have their own advantages and disadvantages, combining the most effective elements of different models could offer childhood cancer survivors the safety of risk-based approaches with patient-orientated solutions that recognize their unique needs in terms of communication, coordination and psychosocial care. Such integrated approaches have the potential to benefit both providers and patients [12].

Understanding the effectiveness of different models is further complicated by the lack of definitive economic analyses, limiting the understanding of the effects on the health system’s efficiency [30]. Only one study in our review demonstrated differences in healthcare utilization for those attending survivorship care compared to those who did not; despite the significant findings in this study, other scholars argue that only those at risk need specialized follow-up as the risk of serious late effects in some cancers is modest [12]. Given these discrepancies, more work is required to inform the most appropriate and sustainable models of care.

Such evidence, including an understanding of cost-shifting is urgently required [5]. Cost shifting may occur when survivors are discharged from one type of service, to then receive care at another. While the goal may be to control costs, it can have unintended consequences on patient flow, resource allocation, and quality of care, which ultimately may not achieve any cost savings to the system as a whole [66].

In future studies, rigorous study designs, including hybrid-design effectiveness-implementation trials that incorporate routinely collected, standardized patient-reported outcomes and elements of care, should be conducted to provide robust evidence and inform service development [67]. Moreover, effectiveness should not be the only concern; the required resources, culture, and environment must also be considered. Implementation studies and hybrid designs offer innovative ways to better understand the barriers and drivers for survivorship care whilst also considering the value proposition.

Two factors further complicate evaluation of models of care: the categorization and descriptions of models and the measurement of outcomes [14, 67]. First, standardizing the nomenclature is crucial and recommendations to describe models include specifying: the lead provider for survivorship care, regularly involved other providers, the location of care, engagement of survivors, location of care, and recipients [67]. These details were lacking in many of the included studies, precluding the ability to draw comparisons between different structure and processes of models of care. Second, comparing outcomes is challenged by the vast array of bio-psychosocial and service-related outcomes, at multiple levels and across a lifespan. The use of a Framework, as employed in this review, assists with organizing outcomes with a logical taxonomy.

### Patient and caregiver experience

Satisfaction from survivor’s perspectives was generally high, regardless of the type of follow-up received, except for some discontent expressed those discharged and receiving no follow-up [59, 68]. Improvements to care models focussed on communication, timeliness and efficiency of information exchange, care coordination, psychoeducation, and late effects awareness [50, 51]. These findings align with the broader literature in this area, emphasizing satisfaction is contingent on quality communication and care [69], which values interpersonal relationships personalized follow-up care [68].

### Barriers to survivorship care

Multiple earlier reviews highlight barriers to childhood cancer survivorship care, contributing to approximately 70% of young survivors lost to follow-up [70, 71]. Barriers exist at the individual, service, and system levels [72], including survivor knowledge of risks [65], lack of primary care provider expertise [73, 74], disparities in equity of access [75], limited capacity within cancer centres [76], and poor communication and coordination between services [77]. Despite the rapidly expanding evidence base of primary studies on childhood cancer survivors, which has quadrupled in the last three decades [78], there remains a notable lack of evidence to address these barriers [79]. Our review reveals a gap not only on understanding the effectiveness of models of care, but the required evidence to demonstrate the impact of survivorship care on medical, psychosocial and health services outcomes [5, 79]. Further work is required to reduce duplication and to understand how interventions, such as the use of SCP, or distance delivered care can contribute to improved outcomes [77]. Additionally as most interventional studies target survivors at the individual level, rather than issues at the provider or systems levels, there remain gaps in understanding the impacts of survivorship services at these levels [3]. Implementation studies are recommended to also address these gaps in knowledge and understanding.

### Strengths and limitations

To our knowledge, this is the first comprehensive review to synthesize a broad range of outcomes for childhood cancer survivorship care models, highlighting diversity in structures and processes and advancing our understanding of this critical aspect of paediatric oncology. This approach, determining quality outcomes, could serve as a benchmark in future studies. Limitations include the possibility of missed relevant studies despite a comprehensive search. We also only included empirical studies published in English; there may be reports of survivorship service outcomes published in the grey literature, or in other languages. Nevertheless, we identified 38 relevant studies, a comparable number to reviews describing quality outcomes for adult cancer survivorship care [18–20].

### Future directions and recommendations

Gaps in evidence highlight areas for future research. Importantly, this review emphasizes that questions regarding the efficacy studies of different models of care are difficult to complete and that other initiatives to advance survivorship care in this population are also needed. Longitudinal designs and implementation studies can provide evidence of sustained effect of interventions or models of care which is desirable in a young population [80]. Additionally, novel study designs that provide robust evidence without the need to randomize individuals, such as hybrid effectiveness trials which also address the barriers to implementation, could advance the field. A gap identified in this review is research that improves outcomes related to health promotion, and identification and management of chronic conditions. Understanding mortality, health service utilization and costs are also required; harnessing data linkage and technology can be used to answer these research questions. Understanding the sustainability of models of care require integration of health economic evaluations in studies. Indeed, evaluating the economic outcomes of different models of care is an understudied area. Standardizing the nomenclature used to describe models of care, and the attributable outcomes would also help with identifying evidence and comparing outcomes. At the policy level, survivorship care needs to be integrated into broader health and social care initiatives. Advocacy for specific resources to support survivorship care across all settings is paramount.

## Conclusion

While the long-term outcomes of childhood cancer survivors may be significantly influenced by the models of care they receive, evidence to date fails to adequately demonstrate an optimal model of care for all childhood cancer survivors. Gaps in understanding regarding elements of care are evident at the individual, provider, and systems level, such as health promotion or chronic disease management and effectiveness in improving health outcomes. Also identified is the need for standardized nomenclature, and economic evaluations to provide a deeper understanding of the efficiency and sustainability of services. The outcomes synthesized in this review provide a valuable reference point for service planning and evaluation, with the ultimate goal of improving long-term outcomes for childhood cancer survivors.

## Supplementary Information

Below is the link to the electronic supplementary material.

Supplementary file1 (DOCX 54 KB)

## Data Availability

Not applicable.
