# Supplementary material for: Childhood cancer models of survivorship care: a scoping review of elements of care and reported outcomes
Source: J Cancer Surviv. 2024 May 9;19(6):1995–2011. doi: 10.1007/s11764-024-01610-6 (PMC12546495; doi:10.1007/s11764-024-01610-6)
Supplement: Supplementary file 1 — Supplementary file1 (DOCX 54 KB) [file 11764_2024_1610_MOESM1_ESM.docx]

## Supplementary Table: Characteristics of included studies

| **Author, Year, Country** | **Study title** | **Study Aims** | **Study design**  **(Level of Evidence)*** | **Model of care** | **Patient age in years**  **Cancer Type**  **Number recruited** | **Main findings** |
| --- | --- | --- | --- | --- | --- | --- |
| Absolom et al.  2006  UK | Predictors of clinic satisfaction among adult survivors of childhood cancer | To describe survivors’ understanding of their late-effects, symptoms, vulnerability, and understanding of the purpose of follow-up and key variables with clinic satisfaction | Cohort with comparator  (Level III) | **Pediatric Cancer Centre vs Adult Cancer Centre Follow-up.** Thirty-minute consultations vs 10 minutes with specialists with expertise in paediatric oncology and adult medicine (endocrinology, haematology, reproductive medicine and psychiatry). Survivors over the age of 16 years routinely transferred. Specialist, when necessary, not formally discharged. | **Age at diagnosis**  7.35 (4.64)  **Age at time of study** 23.29 y (5.53)  Mixed cancers  N=198 | Satisfaction with survivorship care in pediatric and adult follow-up explored, no difference. Reports lower rates of late effects than previous estimates, attributing it to reliance on diagnostic tests and asymptomatic conditions. |
| Alchin et al.  2022  Australia | Childhood Cancer Survivors’ Adherence to Healthcare Recommendations Made Through a Distance-Delivered Survivorship Program | To assess the number of healthcare recommendations accurately recalled by survivors at one and six-months post- intervention | Descriptive observational  (Level IV) | **Multidisciplinary telehealth delivered intervention.**  Survivor previously lost to follow up completes health assessment and provided with recommendations developed by expert MDT | **Age at diagnosis**  < 18 years  **Age at time of study** 31.9,  Mixed cancers,  N=25 | Survivors provided with 6.6 recommendations (range = 1–11) and recalled two at 6 months and adhered to 1.3. 56% did not adhere to any recommendations. Suggests further nurse-led follow-up and education for survivors on late effects prevention strategies. |
| Arpaci et al.  2022  Turkey | ‘Trying to catch up with life’: The expectations and views of adolescent survivors of childhood acute lymphoblastic leukaemia about long-term follow-up care | To describe views, expectations and satisfaction with the LTFU clinic | Qualitative interviews  (Level VI) | **Long Term Follow Up Clinic**  Purpose is to maintain the well-being of the survivors in the long term, to detect late effects earlier and to provide support and consultancy in line with needs. | **Age at diagnosis** 7.37 (3-14),  **Age at time of study** 15.37 (12-19)  Leukaemia  N=16 | Explored information and support needs and preferences of adolescents regarding survival, LTFU, late effects, and healthy habits. Survivors satisfied with care. |
| Arvidson et al.  2006  Sweden | Medical follow-up visits in adults 5-25 years after treatment for childhood acute leukaemia, lymphoma or Wilms’ tumour | To evaluate the need for healthcare, both subjectively and objectively, was met. | Cross Sectional  (Level IV) | **Multiple models**  Most common health provider adult specialist at a hospital (50.0%); 36.4% were still with paediatric specialists, 9.4% had community care visits and 4.2% consulted a private practitioner. | **Age at diagnosis**  < 18 years,  **Age at time of study** 23.5 years, Leukaemia, lymphoma or Wilms,  N=245 | Satisfaction with follow-up visits, highlighting factors influencing attendance. 60% no regular follow-up visits, and 42% of these reported that they missed not having one. 33% dissatisfied with the follow-up programme, 21% had unanswered questions. |
| Aukema et al.  2011  Netherlands | Explorative study on the aftercare of pediatric brain tumor survivors: a parents’ perspective | To describe the need for aftercare for long-term sequelae perceived by parents and to explore the aftercare | Cross Sectional  (Level IV) | **Neuro-oncology After care**  Service from multi-disciplines (not  co-ordinated) including access to educational support, physiotherapy, occupational therapy, and psychology. | **Age at diagnosis**  6.5 (3.3),  **Age at time of study** 14.7 (3.8),  CNS and Brain, N=42 | Noted delays and a lack of parental knowledge about aftercare services. Stresses the need for increased awareness and timely reaction for psychosocial aftercare. |
| Babecoff et al.  2022  Switzerland | Long-term follow-up for childhood cancer survivors: the Geneva experience | To describe the follow-up procedures as well as the chronic health conditions of the LTFU clinic and assess satisfaction of the program | Retrospective Cohort  (Level IV) | **Long Term Follow Up**  Commences after 5/6 years post treatment. Continues until age 21 years. Annual consultation and creation of specific plan adapted to each patient according to medical history. Risk based surveillance in accordance with COG guidelines | **Age at diagnosis**  5 (4.0)  **Age at time of study** 17.7,  Mixed cancers, N=51 | Encourages the maintenance of structured follow-up by a dedicated oncologist for early detection of late side effects. |
| Berger et al.  2017  France | Long-Term Follow-up Consultation After Childhood Cancer in the Rhône-Alpes Region of France: Feedback from Adult Survivors and Their General Practitioners | To evaluate the satisfaction of adult survivors of childhood cancers and their general practitioners (GP) after a long-term consultation. | Descriptive observational  (Level IV) | **Shared care (GP/Specialist)**  Long-term follow-up consultation process that involved joint consultation of the survivor with a pediatric oncologist and an internist. Survivors and GP are sent treatment summaries prior to the joint consultation. | **Age at diagnosis**  4.7  **Age at time of study**, not provided  Mixed cancers, N=150 | Assessed survivor satisfaction with long-term follow-up, emphasizing the positive impact of recommendations provided by clinics. Indicates follow-up consultations, improve survivor and physician knowledge. |
| Carlson et al.  2008  United States | A Multidisciplinary Model of Care for Childhood Cancer Survivors with Complex Medical Needs | To review adherence to long-term follow-up (LTFU) guidelines, assessed provider perspectives, the needs, experience and quality of life child brain tumor survivors | Descriptive observational  (Level IV) | **Multi-disciplinary Survivorship Clinic** Provides same day same access to a range of disciplines and subspecialists and patient appointments are coordinated | **Age at diagnosis**  < 18 years,  **Age at time of study** not reported,  Mixed cancers, N=130 | 110 new problems were identified including endocrine, pulmonary and cardiac conditions. Benefits of the model at the patient level, with reduced time, at the health care provider level, opportunity to provide collaborative care for a complex patient population. |
| Cacciotti et al.  2021  Canada | Late effects care for childhood brain Tumor Survivors: A Quality-Improvement Initiative | To describe a Multidisciplinary Cancer Survivorship Clinic currently in operation at The Children’s Hospital of Philadelphia with a focus on clinic structure, care delivery, and benefits to survivor, health care provider, and institution. | Cross Sectional  (Level IV) | **Long Term Follow Up**  Model focused on assessing healthcare needs and developing a care plan focused on surveillance. | **Age at diagnosis** 8.5,  **Age at time of study** 18.3,  CNS and Brain, N=22 | Explored staff and survivor perceptions of survivorship care, identifying areas for improvement such as increased multi-disciplinary participation and improved patient education. |
| Costello et al.  2017  United States | Shared Care of Childhood Cancer Survivors: A Telemedicine Feasibility Stud | To test feasibility of integrated transition from pediatric oncology care to adult primary care through a collaborative effort between the survivorship team, the PCP, and the survivor, using telemedicine. | Cross Sectional  (Level IV) | **Shared care**  GP and specialist via telemedicine- criteria are >18 years, 10 or more years post cancer treatment, with established GP | **Age at diagnosis** 7.8,  **Age at time of study** 26.52,  Mixed cancers, N=19 | Investigated the impact of telemedicine on patient satisfaction and trust in the provider, suggesting that telemedicine may establish a more trusting relationship. |
| Daly et al.  2019  United States | Survivorship clinic attendance among pediatric and adolescent aged survivors of childhood cancer | To describe the proportion of children and adolescent survivors of childhood cancer who engage in survivorship care at well-established pediatric survivorship clinic two years following the completion of their cancer therapy | Descriptive observational  (Level IV) | **Multi-disciplinary Survivorship Clinic**  A dedicated team of multi-disciplinary specialists with expertise in survivorship. Referred by treating oncologist. Receive a healthcare plan summarising treatment and late effects risk profile | **Age at diagnosis** 8.2.  **Age at time of study** not reported,  Mixed cancers, N=866 | Nearly a third of survivors fail to attend despite the importance of survivor care. Populations at risk include those who received surgery or radiation only, black or mixed race, and those who lived > 25 miles from the clinic. |
| Ducassou et al.  2016  France | Impact of shared care program in follow-up of childhood cancer survivors: An intervention study | To demonstrate that a systematic and organized childhood Cancer Survivor Follow Up Shared Care Plan improves long-term Follow Up. | Cohort with comparator  (Level III) | **Shared care**  Phone appointment between LTFU team and PCP (ii) establishment of a personalized SCP: mandatory clinical consultation by PCP, a hotline was dedicated to PCPs’ queries; (iii) postal confirmation to PCP and patient/family | **Age at diagnosis**  6.2  **Age at time of study** 17.3  Mixed cancers, N=72 | Investigated the impact of a shared care intervention on survivorship care on control and intervention group. Intervention group had higher survey response rates and increased involvement in follow-up by GPs. Supports program but highlights need for nationwide organization, special needs consideration, and sustained funding. |
| Ernst et al.  2022  Germany | A Mixed-Methods Investigation of Medical Follow-Up in Long-Term Childhood Cancer Survivors: What Are the Reasons for Non-Attendance? | To provide insight into German survivors’ care situation, with a particular focus on barriers to follow-up care. | Mixed methods  (Level III) | **Multiple models**  Participants were asked about any kind of medical follow up care they received and the reasons for not attending follow up | **Age at diagnosis** 6.3,  **Age at time of study** 34.92 (5.7),  Mixed cancers,  N= 633 | 50% of participants reported receiving some kind of medical follow-up. Most common reason for non-attendance was lack of information about follow up care and its purpose. 14% had at least 1 chronic health condition. |
| Ford et al.  2013  United States | Attendance at a survivorship clinic: impact on knowledge and psychosocial adjustment | To assess the differences between those who attended a Survivorship Clinic and those who did not on knowledge, perception of risk, and psychosocial adjustment. | Cohort with comparator  (Level III) | **Multi-disciplinary Survivorship Clinic** Attendees at the LTFU were matched with survivors who had never attended for comparison | **Age at diagnosis** 12.6 (6.0)  **Age at time of study** 30.6 (6.1)  Mixed cancers, N=173 | Noted underestimation of future health risks among participants. Explored reasons for non-attendance at clinics among survivors, including lack of awareness and interest. |
| Gandy et al.  2021  USA | “Why We Keep Coming Back”: Family and Provider Perspectives on Factors Influencing Long-term Follow-up for Pediatric Cancer Survivorship Care | To evaluate the barriers to long-term, follow up from patients, caregivers, and their health care providers perspectives | Qualitative interviews  (Level VII) | **Long Term Follow Up Clinic**  The long-term survivorship program offers life-long surveillance and screening for potential late effects associated with cancer, and appointments are typically held annually, or every other year based on the diagnosis, exposures and Children’s Oncology Group guidelines | **Age at diagnosis**  < 18 years,  **Age at time of study** 17,  Mixed cancers, N=10 patients/parents and 8 health professionals | A range of factors influence long-term follow-up for care are described including time off work, school and distance from home. Efficient clinical operations, resources such as parking, provider behaviors, rapport/attachment, and patient/family logistics, institutional operations, their own education and skills. |
| Gebauer et al.  2018  Germany | Multidisciplinary Late Effects Clinics for Childhood Cancer Survivors in Germany – a Two-Center Study | To evaluate the current health status of patients receiving care from collaborative multidisciplinary late effects clinics. | Descriptive observational  (Level IV) | **Long Term Follow Up Clinic**  Outpatients’ clinic at university hospital for yearly long term follow up. Transferred automatically once aged 18 years. Based on their risk of developing late effects, the patients underwent additional examinations | **Age at diagnosis**  8.5 years,  **Age at time of study** 24,  Mixed cancers, N=220 | Investigated the prevalence of chronic health conditions and potential underestimation due to risk stratification. 141 patients (64.1%) suffered from at least 1 chronic health condition and 65 of these (29.6%) were diagnosed with 3 or more health impairments, most commonly endocrine disorders. |
| Harper et al.  2023  Canada | Alberta Childhood Cancer Survivorship Research Program | To quantify the burden of physical and psychosocial late effects, identify risk factors and determine the impact of long-term survivorship clinics at mitigating and preventing late effects. | Retrospective Cohort  (Level III) | **Multi-disciplinary Survivorship Clinic** led by a dedicated pediatric oncologist with expertise in late effects. Follow survivors from 2 years post therapy into adulthood. Model include use of a templated Survivorship Care Plan with treatment history and advice and guidance for patient specific LTFU | **Age at diagnosis** 8.1,  **Age at time of study** 19.7,  Mixed cancers, N=1379 | Presented findings from a research program involving a large cohort of childhood cancer survivors, aiming to inform and improve clinical care. 82 secondary neoplasms during study period, 437 deaths. Inpatient and ambulatory care events reported- but may not relate to post treatment phase |
| Haupt et al.  2018  Italy | The ‘Survivorship Passport’ for childhood cancer survivors | To report outcomes from the Survivorship Passport (SurPass) study. | Cross Sectional  (Level IV) | **Survivorship Care Plans**  The Survivorship Passport (SurPass) consists of electronic documents, provides a summary, together with personalised follow-up and screening recommendations based on guidelines published by the International Guidelines Harmonization Group and PanCareSurFup | **Age at diagnosis** not reported,  **Age at time of study** 17.1,  Mixed cancers, N=190 | Explored the impact of SurvivorPass delivery on survivors, noting both positive and negative effects, including increased anxiety in some. The SurPass, at the institutional, regional or national level deemed a sustainable solution for national healthcare systems to systematically organise LTFU care in a consistent and cost-effective way. |
| Kam et al.  2016  Canada | Evaluation of a joint adult and pediatric clinic for cancer survivorship care | To describe the care experience of young adult survivors of childhood cancer at a joint adult/pediatric After Care program. | Cross Sectional  (Level IV) | **Joint Pediatric/Adult Aftercare clinic**  The long-term follow-up care through The Aftercare clinic is delivered through a team approach where Pediatric and Adult oncologists work together to provide continuity of care for adult survivors of childhood cancer. | **Age at diagnosis** 8.5,  **Age at time of study** 23.8,  Mixed cancers, N=73 | High attendance, with low loss to follow-up. 5-year loss to follow up 3.8%. Participants described a positive cancer care experience but reported a high rate of cancer-related worries but good self-management skills |
| Kadan-Lottick et al.  2018  USA | Randomized Trial of the Impact of Empowering Childhood Cancer Survivors with Survivorship Care Plans | To evaluate the impact of randomly assigning childhood cancer survivors to 1) SCPs to be taken to their primary care physician (PCP) to implement or on health care quality measures. | Randomised controlled trial  (Level II) | **Survivorship care plan** randomised to implement with **Primary Care** or **Survivorship clinic**. Assessed adherence to guidelines and numbers of new late complications | **Age at diagnosis**  9.8,  **Age at time of study** 15.9,  Mixed cancers, N=96 | Adherence to guideline recommended surveillance ranged 0-47% in PCP group and 50-86% in survivorship clinic. Complications in PCP group (2.1 v 23%). Delivering a SCP to childhood cancer survivors with directions to follow-up with their PCPs is not sufficient to achieve high quality survivorship care. |
| Keats et al.  2019  Canada | After Childhood Cancer: A Qualitative Study of Family Physician, Parent/Guardian, and Survivor Information Needs and Perspectives on Long-Term Follow-up and Survivorship Care Plans | To examine perspectives of a novel personalized SCP for childhood cancer survivors, their family, and family physicians. | Qualitative interviews  (Level VI) | **Primary Care Provider**  Follow up supported by individual needs, preferences, and perceived utility of a personalized, algorithm-driven, automatically generated SCP to use in transition to FP care | **Age at diagnosis**  9 (4.7),  **Age at time of study** 23 (5.6),  Mixed cancers, N=8 patients and 6 health professionals | Examined the value of Survivorship Care Plans to enhance patient and family understanding. Barriers include lack of confidence in the Primary Care Provider knowledge and concerns about transfer of information as the child becomes an adult. |
| Knapke et al.  2012  USA | Hereditary Cancer Risk Assessment in a Pediatric Oncology Follow-Up Clinic | To quantify eligibility for genetics’ referrals from a long-term survivor clinic, as well as characterize the features of the clinical or family history which warrant eligibility. | Descriptive observational  (Level IV) | **Multi-disciplinary Survivorship Clinic**  A multidisciplinary team of healthcare providers is available to survivors and families including the addition of a certified genetic counselor. | **Age at diagnosis** not reported,  **Age at time of study** 18,  Mixed cancers, N=370 | Explored the impact of adding a certified genetic counsellor to the Cancer Survivor Clinic, significantly increasing the percentage of patients eligible for genetics evaluation. 29% were considered eligible for genetics follow-up or referral. Supports inclusion of genetic evaluation in the care of childhood cancer survivors |
| Lie et al.  2017  Norway | Experiences with late effects-related care and preferences for long-term follow-up care among adult survivors of childhood lymphoma | To explore the experiences with late effects-related care among adult survivors of childhood lymphomas and preferences for long-term follow-up care. | Qualitative interviews  (Level VI) | **Shared care**  Survivors discharged from routine follow up care to Primary Care Provider in collaboration with other specialists | **Age at diagnosis**  13,  **Age at time of study** 37,  Mixed cancers, N=34 | Highlighted negative experiences with late effects-related care among survivors. Emphasized the need for increased knowledge dissemination beyond oncology. Advocates for well-designed shared care models involving specialists, GPs, and survivors. |
| Lindell et al.  2015  US | Knowledge of Diagnosis, Treatment History, and Risk of Late Effects Among Childhood Cancer Survivors and Parents: The Impact of a Survivorship Clinic | To quantify the impact of specialized survivorship care on survivor and parent knowledge between control group and group attending survivorship clinic | Cohort with retrospective comparator  (Level III) | **Nurse-led survivorship clinic**  Provides adjunct care to pediatric oncologist. Educational booklet provided individualised to diagnosis and treatment | **Age at diagnosis** 6.8,  **Age at time of study** not reported,  Mixed Cancers, N=174 | Those in nurse led clinic more likely compared to controls to know diagnosis (98 v 90%), remember discussion of risk of late effects (99 v 62%). Nurse led clinic associated with 🡩 knowledge of risks, although this decreased over time. |
| Linendoll et al.  2021  USA | The Creation of a Comprehensive Adolescent and Young Adult Cancer Survivorship Program: ‘‘Lost in Transition’’ No More | To describe the process of establishing the dedicated survivorship clinic, including the creation of a database on cancer history, exposures, and attendant risks of late effects. | Descriptive observational  (Level IV) | **Multi-disciplinary Survivorship Clinic** A comprehensive, academic, longitudinal model created to provide care to 18–39-year-olds with a history of cancer, regardless of their age at diagnosis. Long-term follow-up care is based on individual treatment exposures and guideline-based associated risks. | **Age at diagnosis** majority < 14 years, **Age at time of study** 26,  Mixed cancers, N=144 | Reports addressing both physical and mental health aspects in survivors. Associates survivorship care with increased knowledge of diagnosis, treatment history, and late effects risk. Two-thirds had an established late effect, one third with an established medical comorbidity, and 11% with secondary cancer related to their oncologic treatment. 32 % had a known affective disorder with one quarter already taking a psychotropic medication. 85% of patients remained in long-term follow-up clinical model. |
| Marr et al.  2017  Canada | Specialized survivor clinic attendance increases adherence to cardiomyopathy screening guidelines in adult survivors of childhood cancer | To determine if increased utilization of the clinics was associated with improved adherence to the recommended schedule of echocardiography testing. | Retrospective Cohort  (Level IV) | **Long Term Follow Up Clinic**  ~5 years after diagnosis pediatric patients are transferred to a specialized survivor clinic at their pediatric cancer center. The COG LTFU guidelines are adopted in all clinics as a framework for surveillance recommendations | **Age at diagnosis**  < 18 years,  **Age at time of study** not reported,  Mixed cancers, N=1811 | Associated increased utilization of survivor clinics with improved patient attendance and adherence compared to those that don’t attend (RR 10.6 v 3.3). 30% reported annual physical exam with GP. Specialized survivor clinics may improve health outcomes in survivors through improved adherence to screening. |
| Mayes et al.  2016  UK | Health promotion and information provision during long-term follow-up for childhood cancer survivors: A service evaluation | To ascertain teenage and young adult survivors’ experiences and views on the provision of information regarding future health risks, disease prevention, and health promotion. | Qualitative interviews  (Level VI) | **Long Term Follow Up Clinic**  ~ 5 years after treatment and are most seen annually. Patients informed of future health risks and impacts of adverse health behaviours | **Age at diagnosis** 11.3,  **Age at time of study** 21.8,  Mixed cancers, N=51 | Explored childhood cancer survivors' satisfaction with information received about late effects. Noted varying expectations about the purpose of long-term follow-up (LTFU) and a desire for more details. |
| Meeske et al.  2007  USA | Factors associated with health-related quality of life in pediatric cancer survivors | To describe HRQOL in pediatric cancer survivors attending a long-term follow-up clinic and to identify demographic and disease/treatment-related factors associated with poor quality of life outcomes. | Cross Sectional  (Level IV) | **Long Term Follow Up Clinic**  Long-term information, follow-up, and evaluation (LIFE) clinic at Childrens Hospital Los Angeles | **Age at diagnosis**  4 (2.6),  **Age at time of study** 13.3 (2.9),  Mixed cancers, N= 86 | Health-Related Quality of Life among pediatric cancer survivors, differed based on based on cancer type. Brain tumor diagnosis, non-Caucasian ethnicity, and poorer psychosocial functioning 🡫 QoL Suggests routine administration of standardized measures for assessing the need for detailed psychosocial assessment. |
| Mellblom et al.  2015  Norway | Providing information about late effects during routine follow-up consultations between pediatric oncologists and adolescent survivors: a video based, observational study | To investigate to what extent potential late effects were discussed and information provided, to adolescent and young adult (AYA)-aged survivors (of pediatric cancer) during routine follow-up consultations. | Descriptive observational  (Level IV) | **Long Term Follow Up Clinic**  Routine follow-up consultations between survivors of pediatric cancer and pediatric oncologists | **Age at diagnosis** 6.7,  **Age at time of study** 15.3,  Mixed cancers, N=66 | Potential late effects discussed in 85% of the consultations. Of these, 71% were initiated by the oncologist, and 60% concerned existing health problems. The observed oncologist variability in providing such information indicates a need for standardization of information practices. Noted a mismatch between survivors' desired topics of discussion and what was covered in appointments. |
| Michel et al.  2009  England | Follow-up care after childhood cancer: Survivors’ expectations and preferences for care | To describe (1) self-rated quality of life, late effects and perceived future vulnerability, (2) expectations before a follow-up appointment, subsequent satisfaction and preferences for different models of care. | Descriptive observational  (Level IV) | **Multiple models**  Postal/telephone follow up, Primary care provider led, nurse-led, and consultant led clinic with input from an oncologist, endocrinologist and late effects specialist nurse. Reproductive specialist and psychiatric support are available if needed. | **Age at diagnosis**  7 (4.5),  **Age at time of study** 28.2 (7.0),  Mixed cancers, N=112 | Childhood cancer survivors are in favour of sustaining long-term follow-up care within the existing consultant-led model, but this is not feasible given the increasing number of survivors. Physical and psychological quality of life similar to that of population norms. |
| Northman et al.  2015  USA | Supporting Pediatric Cancer Survivors with Neurocognitive Late Effects: A Model of Care | To provide an overview of the SLP model of care and discuss parent-perceived quality and effectiveness. | Descriptive observational  (Level IV) | **School liaison program**  As patients transition off of active treatment, referred to School Liaison Program services. Following assessment, psychoeducation is provided to parents and schools. Advocacy for appropriate educational supports and services to enable child to reach academic potential. | **Age at diagnosis**  < 18 years,  **Age at time of study** not reported,  Mixed cancers, N=57 | Parents attributed School liaison involvement to improved academic performance, home-school communication, and school-level understanding of unique student cognitive profiles and learning needs. |
| Ou et al.  2017  USA | An investigation of survivorship clinic attendance among childhood cancer survivors living in a five-state rural region | To examine how demographic, clinical, and geographic-based characteristics are associated with attendance at the only pediatric survivorship clinic. | Retrospective Cohort  (Level III) | **Multi-disciplinary Survivorship Clinic** Typically referred 5 years after diagnosis, eligible patients did not need surveillance imaging more than once per year. | **Age at diagnosis**  11,  **Age at time of study** 21,  Mixed cancers, N=1812 | Identified low (5%) attendance of childhood cancer survivors at survivorship clinics, especially in rural areas. Attributed differences to factors such as insurance accessibility, geographic scope, and clinic scheduling protocols |
| Pannier et al.  2019  USA | Survivorship care plan experiences among childhood acute lymphoblastic leukemia patients and their families | To determine parent and patient experiences with receiving a survivorship care plan and to identify their preferences for survivorship care plan delivery. | Cross Sectional  (Level IV) | **Survivorship Care Plans**  Template similar content as Passport for Care’s SCP, with additional information on vaccinations and health behaviors per feedback. QR codes that link to Children's Oncology Group follow-up guidelines. | **Age at diagnosis**  ≤14 years, Age at time of study > 18 years,  Leukaemia,  N=21 | Explored parent satisfaction with Survivorship Care Plans (SCP), 70% thought the SCP should be delivered after treatment but by T3 most preferred the plan to be delivered before the end of treatment (60.9%). While 95.7% of parents intended to share their child’s SCP with another provider, family, or school at T2, only 60.9% had done so by T3 (P < 0.01). At both T2 and T3, 100% of parents agreed that the SCP would help make decisions about their child’s future health care. Most patients at T3 (83.3%) felt they had learned something new from their SCP. |
| Reynolds et al.  2019  Canada | A comparison of two models of follow-up care for adult survivors of childhood cancer | To determine whether ASCCs in one model would report lower quality of life (QOL) and more cancer-related physical symptoms compared to ASCCs in the other model. | Cohort with comparator  (Level III) | **Long Term Follow Up Clinic** compared with **Primary Care Model**.  LTFU service patients 2 years after treatment with no age limit. Primary Care Model serves patient 2 years off treatment until age 20 years or 10 years after treatment (whichever is last). | **Age at diagnosis** 7.16 (4.85),  **Age at time of study** 29.08 (4.9),  Mixed cancers, N=156 | Favoured LTFU model because of significant differences in adherence to recommended follow-up tests (85 v 29%) and fewer (0.4-7.1%) symptoms. Half of the parents showed a lack of knowledge about aftercare services. No difference in QOL |
| Ross et al.  2019  USA | Impact of Survivorship Care on Young Adult Survivors of Childhood Cancer with Post-Traumatic Stress Symptoms | To describe the severity and frequency of PTSS in a sample of young adult survivors who had not previously attended a survivorship clinic | Randomised controlled trial  (Level II) | **Multi-disciplinary Survivorship Clinic**  As part of a larger trial, participants who had not previously attended survivorship care were randomized to a model of survivorship-focused health care. | **Age at diagnosis** 14.8,  **Age at time of study** 21.5,  Mixed cancers, N=44 | Explored post-traumatic stress symptoms (PTSS) in young adults attending survivorship clinics. Almost 50% were classified as partial or likely PTSD likely or PTSD likely. Elevated symptoms included avoidance or numbing. At follow-up, PTSSs did not differ significantly from baseline. Found considerable rates of PTSS but emphasized that survivorship care did not trigger or aggravate these symptoms. |
| Sutradhar et al.  2015  Canada | Specialized survivor clinic attendance is associated with decreased rates of emergency department visits in adult survivors of childhood cancer. | To determine whether prior attendance at survivor clinics by adult survivors of childhood cancer was associated with rates of emergency department visits. | Cohort with comparator  (Level III) | **Multi-disciplinary survivorship clinic**  Launched in 1999, specialised survivor clinics available to those who have completed treatment in Canada | **Age at diagnosis**  9 years,  **Age at time of study** not reported,  Mixed cancers, N=3912 | Individuals who had at least 1 prior visit to a survivor clinic had a 19% decreased rate of ED visits in comparison with individuals who had not visited a survivor clinic (adjusted relative rate, 0.81; 95% confidence interval, 0.78-0.85). Each additional prior visit to a survivor clinic was associated with a 5% decrease in the rate of ED visits (adjusted relative rate, 0.95; 95% confidence interval, 0.93-0.96). |
| Vetsch et al.  2016  Switzerland | Follow up care of young cancer survivors: attendance and parental involvement | To describe the current follow-up care of young childhood cancer survivors in Switzerland, including specialists visited and reasons for non-attendance. | Descriptive observational  (Level IV) | **Long Term Follow Up Clinic**  Followed-up by their pediatric oncologist for 10 years after diagnosis often into their early twenties and then usually discharged to a general practitioner (GP) or medical oncologist. | **Age at diagnosis** 3.4,  **Age at time of study** 14.7,  Mixed cancers, N=189 | Emphasized parental involvement in medical visits for young children, particularly in motivating children to stay in follow-up. Links lower follow-up attendance to longer duration since diagnosis. Advocates for educating survivors and parents on the importance of follow-up care. |
| Whitaker et.al  2021  USA | Neuropsychological surveillance model for survivors of pediatric cancer: A descriptive report of methodology and feasibility | To describe this integrated neuropsychological monitoring and triage service over the first three years of implementation. | Descriptive observational  (Level IV) | **Neuropsychologists follow up**  Regardless of perceived deficiency, all survivors screened and referred to neuropsychology if warranted | **Age at diagnosis**  5.6 (4.1),  **Age at time of study** 13.2 (4.3),  Mixed cancers N=215 | Emphasized the need for broad-based neuropsychological care for survivors of childhood cancer, highlighting current limitations in access. 25% of those screened required further assessment |

LTFU= Long Term Follow Up; SCP= Survivorship Care Plan; QOL= Quality of Life; PCP= Primary care Provider

*Levels of Evidence (LoBiondo-Wood & Haber, 2002):

Level I: Systematic reviews or meta-analysis of randomised controlled trials, evidence-based clinical practice guidelines

Level II: Well-designed randomised controlled trials

Level III: Controlled trial without randomisation or quasi experimental study

Level IV: Single nonexperimental study, e.g. case control, correlational, cross sectional, cohort study

Level V: Systematic review and qualitative studies

Level VI: single descriptive or qualitive study

Level VII: Opinion of authority and/or expert committees

LoBiondo-Wood, G., & Haber, J. (2002). Nursing research: Methods and critical appraisal for evidence-based practice.

## Supplementary File 2

## Search Strategy example MEDLINE via EBSCO

Population: Child OR infant AND cancer survivor

S1 children OR kids OR youth Or Child OR Expand- equivalent subjects

S2 pe*diatric OR infant Expand- equivalent subjects

S3 #S1 OR #S2

S4 Cancer survivors Expand- equivalent subjects

Interventions: Follow up care OR aftercare OR model of care OR guidelines

S5 follow up care OR followup OR aftercare OR

model of care OR guidelines Expand- equivalent subjects

Outcomes: Health outcomes, implementation outcomes (domains of care, contextual domains), economic outcomes

S6 outcomes OR benefits OR effects OR impact OR

effectiveness OR outcome evaluation Expand- equivalent subject

Strategy:

#S3 AND #S4 AND #S5 AND #S6

**S1 children**: "child"[MeSH Terms] OR "child"[All Fields] OR "children"[All Fields] OR "child's"[All Fields] OR "children's"[All Fields] OR "childrens"[All Fields] OR "childs"[All Fields] OR "child"[MeSH Terms] OR "child"[All Fields] OR "children"[All Fields] OR "child's"[All Fields] OR "children's"[All Fields] OR "childrens"[All Fields] OR "childs"[All Fields]

**S2 infant**: "infant"[MeSH Terms] OR "infant"[All Fields] OR "infants"[All Fields] OR "infant's"[All Fields]

**S4 cancer survivor**: "cancer survivors"[MeSH Terms] OR ("cancer"[All Fields] AND "survivors"[All Fields]) OR "cancer survivors"[All Fields] OR ("cancer"[All Fields] AND "survivor"[All Fields]) OR "cancer survivor"[All Fields]

**S5 follow up care**: "aftercare"[MeSH Terms] OR "aftercare"[All Fields] OR ("follow"[All Fields] AND "up"[All Fields] AND "care"[All Fields]) OR "follow up care"[All Fields] OR "followup"[All Fields] OR "followups"[All Fields]

**Aftercare:** "aftercare"[MeSH Terms] OR "aftercare"[All Fields]

**model:** "model"[All Fields] OR "model's"[All Fields] OR "modeled"[All Fields] OR "modeler"[All Fields] OR "modeler's"[All Fields] OR "modelers"[All Fields] OR "modeling"[All Fields] OR "modelings"[All Fields] OR "modelization"[All Fields] OR "modelizations"[All Fields] OR "modelize"[All Fields] OR "modelized"[All Fields] OR "modelled"[All Fields] OR "modeller"[All Fields] OR "modellers"[All Fields] OR "modelling"[All Fields] OR "modellings"[All Fields] OR "models"[All Fields]

**S6 outcome:** "outcome"[All Fields] OR "outcomes"[All Fields]

**benefit:** "benefit"[All Fields] OR "benefited"[All Fields] OR "benefiting"[All Fields] OR "benefits"[All Fields] OR "benefitted"[All Fields] OR "benefitting"[All Fields]

**effect:** "effect"[All Fields] OR "effecting"[All Fields] OR "effective"[All Fields] OR "effectively"[All Fields] OR "effectiveness"[All Fields] OR "effectivenesses"[All Fields] OR "effectives"[All Fields] OR "effectivities"[All Fields] OR "effectivity"[All Fields] OR "effects"[All Fields]

**impact:** "impact"[All Fields] OR "impactful"[All Fields] OR "impacting"[All Fields] OR "impacts"[All Fields] OR "tooth, impacted"[MeSH Terms] OR ("tooth"[All Fields] AND "impacted"[All Fields]) OR "impacted tooth"[All Fields] OR "impacted"[All Fields]

**effectiveness:** "effect"[All Fields] OR "effecting"[All Fields] OR "effective"[All Fields] OR "effectively"[All Fields] OR "effectiveness"[All Fields] OR "effectivenesses"[All Fields] OR "effectives"[All Fields] OR "effectivities"[All Fields] OR "effectivity"[All Fields] OR "effects"[All Fields]

**outcome:** "outcome"[All Fields] OR "outcomes"[All Fields]

evaluation: "evaluability"[All Fields] OR "evaluate"[All Fields] OR "evaluated"[All Fields] OR "evaluates"[All Fields] OR "evaluating"[All Fields] OR "evaluation"[All Fields] OR "evaluation's"[All Fields] OR "evaluations"[All Fields] OR "evaluative"[All Fields] OR "evaluatively"[All Fields] OR **"evaluatives**"[All Fields] OR "evaluator"[All Fields] OR "evaluator's"[All Fields] OR "evaluators"[All Fields]

Supplementary File 3 Eligibility Criteria

|  | Inclusion | Exclusion |
| --- | --- | --- |
| Population | Reports on more than 50% of the population underage 14 years at diagnosis  OR  able to obtain outcomes measures for age groups of interest:  Child (0-14yrs)  Cancer survivor post end of treatment | Report includes less than 50% of the population under age 14 years at diagnosis  No outcome measures available for age groups of interest  Time since treatment not defined, or able to be identified  Receiving palliative care |
| Intervention | Cancer survivorship is the focus of the paper  Describes provision or process of follow-up care OR models of care including risk stratification. E.g. supported self-management  Shared care; multi-disciplinary clinical care, nurse-led models of care | Interventional or experimental studies that do not include model of care  No description of how care is organised or delivered |
| Outcomes | **Health outcomes**: health-related quality of life, mortality morbidity, cancer-related late-effects and second neoplasms/ recurrence  **Domains of care:** processes that evaluate prevention/ surveillance of cancer; processes that describe assessment and management of physical and psychological effects; processes that describe assessment and management of chronic medical conditions, late effects health promotions and disease prevention  **Contextual domains:** description of clinical structure, communication decision making, care coordination, patient and family experience. Reports of barriers and facilitators to implementation of follow-up care.  **Economic outcomes:** healthcare utilization; costs of care | No outcomes of interest reported |
| Study design | All study designs, quantitative and qualitative will be included.  Systematic reviews will be considered if there is an explicit aim and systematic processes | Literature reviews  Opinion pieces, commentaries, unpublished work, editorials, letters, conference proceedings |
